# Identification and characterization of dynamically regulated hepatitis-related genes in a concanavalin A-induced liver injury model

**DOI:** 10.18632/aging.104089

**Published:** 2020-11-18

**Authors:** Anna Chen, Yidong Wang, Jiaqi Wu, Dong Tang, Qianru Zhu, Anqian Lu, Jin Yang, Zhejun Cai, Junping Shi

**Affiliations:** 1 Translational Medicine Center, The Affiliated Hospital of Hangzhou Normal University, Hangzhou 310015, China; 2 Department of Cardiology, The Second Affiliated Hospital, Zhejiang University School of Medicine, Hangzhou 310009, China; 3 Department of Radiology, The Affiliated Hospital of Hangzhou Normal University, Hangzhou 310015, China; 4 Institute of Hepatology and Metabolic Diseases, Hangzhou Normal University, Hangzhou 310015, China

**Keywords:** autoimmune hepatitis, concanavalin A, Cd63, transcriptome

## Abstract

Background: Concanavalin A (ConA)-induced liver damage of mice is a well-established murine model mimicking the human autoimmune hepatitis (AIH). However, the pathogenic genes of the liver injury remain to be revealed.

Methods: Using time-series liver transcriptome, top dynamic genes were inferred from a set of segmented regression models, and cross-checked by weighted correlation network analysis (WGCNA). AIH murine models created by ConA were used to verify the *in vivo* effect of these genes.

Results: We identified 115 top dynamic genes, of which most were overlapped with the hub genes determined by WGCNA. The expression of several top dynamic genes including *Cd63, Saa3, Slc10a1, Nrxn1, Ugt2a3,* were verified *in vivo*. Further, Cluster determinant 63 (*Cd63*) knockdown in mice treated with ConA showed significantly less liver pathology and inflammation as well as higher survival rates than the corresponding controls.

Conclusion: We have identified the top dynamic genes related to the process of acute liver injury, and highlighted a targeted strategy for *Cd63* might have utility for the protection of hepatocellular damage.

## INTRODUCTION

Autoimmune hepatitis (AIH) is a chronic and progressive inflammatory liver disease with a prevalence of 15 cases per 100,000 individuals worldwide [1]. Currently, corticosteroids and azathioprine are used as standard therapy of AIH patients [2]. However, 10-20% of AIH patients are refractory to corticosteroids or azathioprine and progress to cirrhosis and end-stage-liver disease [3]. In the absence of any treatment, nearly 50% of patients with severe AIH die within approximately 5 years [4]. Hence, there is an urgent need to identify genes that correlate with AIH pathogenesis for development of targeted therapy to improve survival outcomes.

Genetic susceptibility, circulating autoantibodies, molecular mimicry, and immune disorders, including dysfunctional T-lymphocyte activation are all related to AIH pathogenesis [5, 6]. However, reliable experimental animal models are required to unravel the mechanistic details underlying AIH and test candidate drugs to alleviate AIH [7, 8]. Concanavalin A (ConA) is a plant lectin that binds to sugar residues of extracellular proteins, thereby agglutinating blood erythrocytes and stimulating immune cells, especially T-lymphocytes [9]. Concanavalin A (ConA)-induced hepatitis model mice mimic AIH characteristics and have been used to evaluate the activity of AIH candidate drugs [10]. However, the genes that regulate ConA-induced liver injury have not been evaluated.

High-throughput genome-wide transcriptome profiling is commonly to identify changes in gene expression and biological pathways under various physiological, pathological or specifically ordered conditions over time or space [11, 12]. Therefore, in this study, we analyzed the liver transcriptome data in the mouse model of ConA-induced hepatitis using Trendy and WGCNA to identify critical genes associated with liver pathology.

## RESULTS

### Identifying dynamic gene expression changes during concanavalin A-induced acute liver injury using Trendy

**Figure 1:**
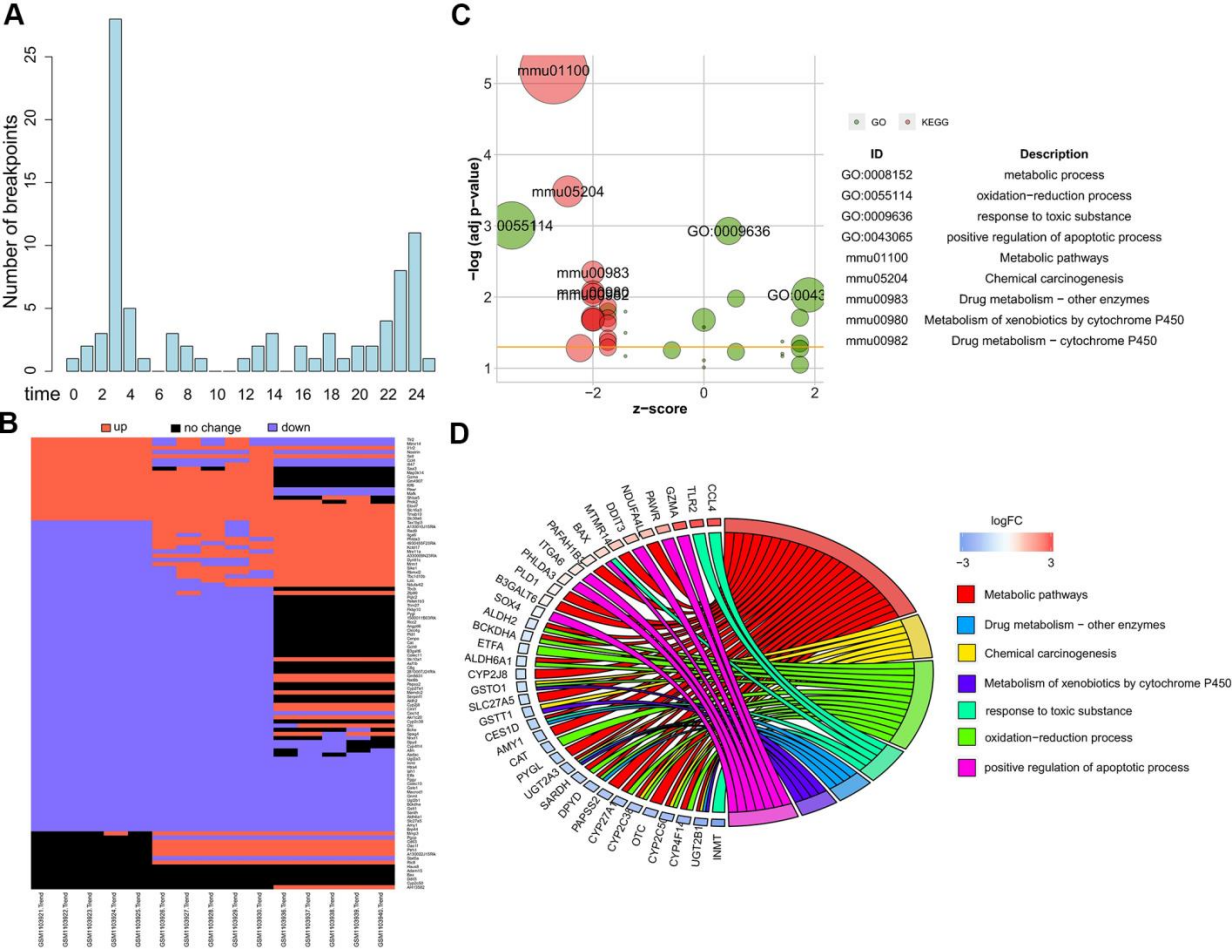
**Dynamics of global gene expression after ConA treatment.** (**A**) The breakpoint distribution of 115 top dynamic genes identified by Trendy with R^2^ > 0.98 is shown. (**B**) Heatmap shows the expression of top dynamic genes in the liver samples harvested at 0h, 3h, and 24h from 10 mg/Kg ConA treated mice (GSE45413). (**C**) The most significantly enriched GO terms and KEGG pathways for the top dynamic genes are shown. (**D**) The GO Chord plot shows the enriched biological function terms for the top dynamic genes. The genes are listed on the left side and their fold change values are shown according to the color scale.

We used the Trendy software to analyze global gene expression changes in liver tissues from ConA-induced hepatitis model mice and identify genes with breakpoints/segments (upregulation or downregulation) at 3 h and 24 h after ConA treatment. We identified 115 top dynamic genes with an adjusted R^2^ >0.98 (Supplementary Table 1). We observed breaks or changes in gene expression at 3 h and 24 h after ConA treatment in all the top dynamic genes compared to their corresponding gene expression at the 0 h time point (Figure 1A). This showed at least two time points or segments at which the top dynamic genes showed differential expression in response to ConA treatment. The differential expression patterns (upregulation or downregulation) of all the top dynamic genes by combining both the time points together are shown in Figure 1B.

Next, we performed functional enrichment analysis of the top dynamic genes to determine the plausible pathogenetic mechanisms underlying ConA-induced acute liver injury. The most significantly enriched gene ontology (GO) terms related to the top dynamic genes were metabolic process, oxidation-reduction process, response to toxic substances, and positive regulation of apoptotic process (Figure 1C, 1D). The most significantly enriched KEGG pathways were metabolic pathways, chemical carcinogenesis and drug metabolism (Figure 1C). Peng et al. showed that exposure of hepatic cells to toxic substances induced chronic inflammation-related dynamic changes in metabolism [13]. Therefore, we postulate that the top dynamic genes dysregulate metabolic pathways in the ConA-induced hepatitis model mice.

### Verification of the top dynamic genes in the ConA-induced hepatitis model mice

We established the ConA hepatitis model mice as shown in Supplementary Figure 1. ConA-treated mice showed significantly enlarged liver, spleen and kidneys and elevated serum ALT and AST levels upon ConA treatment. H&E stained liver sections of ConA-treated mice showed significant infiltration of inflammatory cells, massive hepatocyte necrosis, and disordered hepatic sinusoid structures (Supplementary Figure 1). These findings demonstrated AIH-like characteristics in the ConA hepatitis model mice.

**Figure 2:**
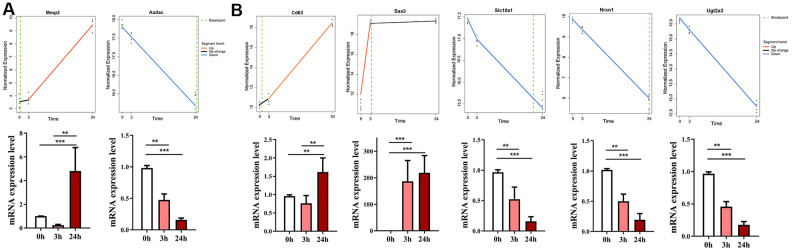
**Validation of gene expression of top dynamic genes in the ConA hepatitis model mice.** (**A**) The expression of two genes with the earliest or latest breakpoint time (*Mmp3* at 3 h, and *Aadac at* 24 h) as determined by Trendy (upper panel) were verified by qRT-PCR analysis (bottom panel) of the liver tissues from the *in vivo* ConA-treated hepatitis model mice. (**B**) qRT-PCR results show the mRNA levels of the top 5 dynamic genes (*Cd63, Saa3, Slc10a1, Nrxn1, Ugt2a3*) in the liver tissues of the ConA-liver injury model mice at 0h, 3h, and 24 h respectively. All data are shown as means ± SEM (n = 5 per group). * *p* < 0.05; ** *p* < 0.01; *** *p* < 0.001.

Next, we examined the gene expression of two candidate genes having the extreme breakpoints identified by Trendy, namely, matrix metallopeptidase 3 (*Mmp3*) and arylacetamide deacetylase (*Aadac*) in the liver tissues of ConA-induced hepatitis model mice. Trendy analysis showed that the expression of *Mmp3* significantly increased at 3 h and peaked at 24 h in the livers of ConA-treated mice (Figure 2A). The mRNA expression of *Aadac* significantly decreased in the ConA-treated mice livers at 24 h post-ConA treatment (Figure 2A). Both *Mmp3* and *Aadac* play significant roles in the liver functions [14]. *Aadac* is involved in lipolysis of cellular triacylglycerol stores and the assembly of very low-density lipoprotein (VLDL) [15]. The top 5 dynamic genes (*Cd63, Saa3, Slc10a1, Nrxn1, Ugt2a3*) differentially expressed in the ConA-treated mice livers were verified by qRT-PCR analysis (Figure 2B). These results confirm that significant changes in the expression of the top dynamic genes correlate with acute hepatitis in the ConA-induced hepatitis model mice.

### WCGNA identifies two top modules with hub genes that overlap with top dynamic genes identified by Trendy

**Figure 3:**
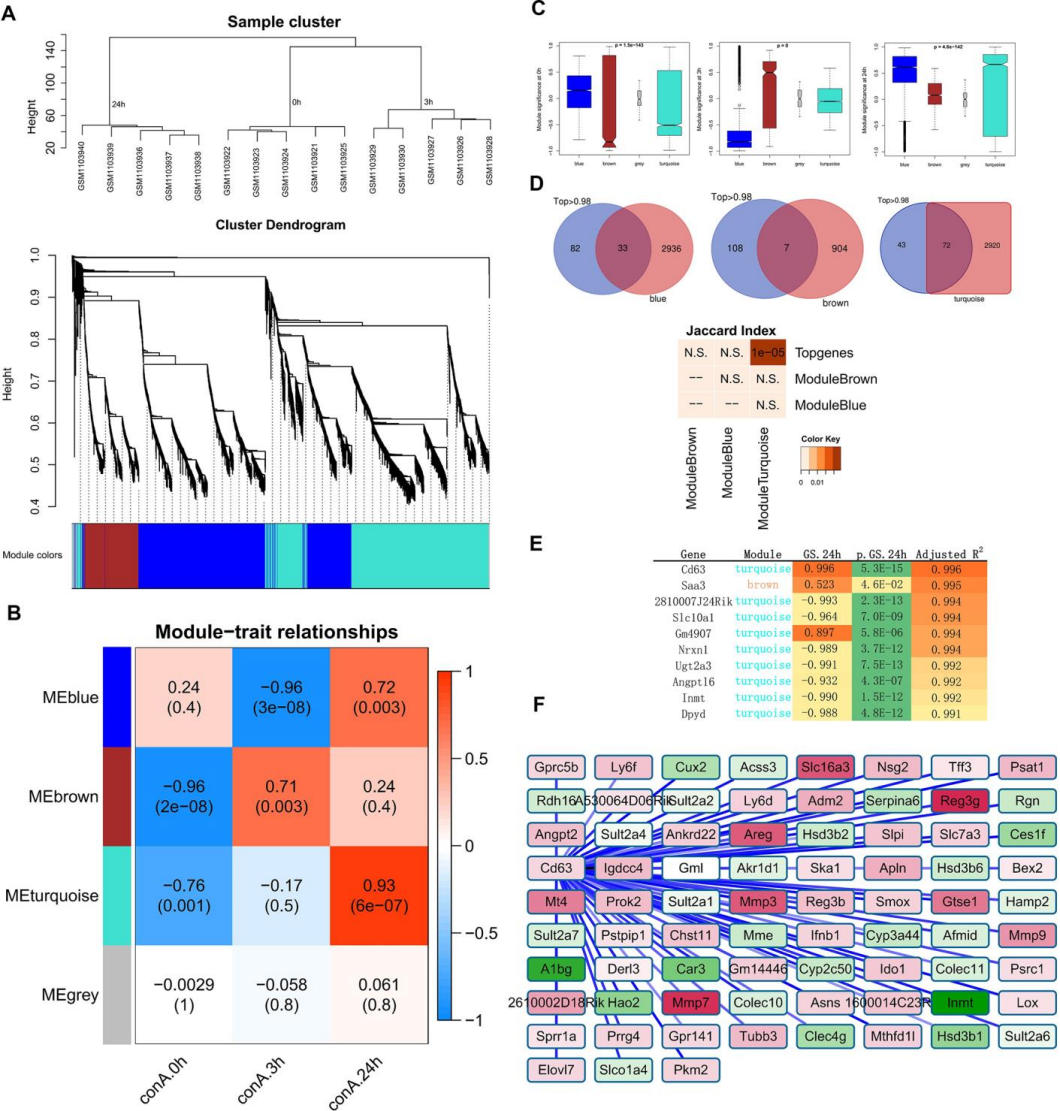
**Identification of top gene modules and hub genes related to liver injury in ConA-treated mice by WGCNA.** (**A**) The cluster dendrogram of 6936 genes in the ConA-treated murine liver samples is shown. The colored bars at the bottom show the color that are designated for specific gene clusters (3 modules). (**B**) Heatmap shows the correlation between module eigengenes (ME) and the trait (time of liver injury). Each row corresponds to a single module eigengene and the corresponding column represents a trait. Each cell contains the corresponding correlation and p value. (**C**) Module significance (MS) of each module based on the average absolute gene significance values of all genes in a module are shown for the 3 h and 24 h time points. (**D**) Venn diagram shows the overlap between the top genes identified by Trendy and the module genes detected by WGCNA. The lower panel shows the significant overlap between the top dynamic genes and the module genes in the turquoise module. (**E**) Top 10 hub genes in the turquoise module and their gene significance values. (**F**) Gene co-expression network shows the relationship between *Cd63* and its co-expressed genes. The node colors are denoted from green to red (low to high) based on the fold change of gene expression between 3-24 h after ConA administration relative to their expression at 0h.

Weighted gene co-expression network analysis (WGCNA) was widely utilized to identify the hub genes of diseases. To determine if the top dynamic genes identified by Trendy could also be reproduced by the hub gene analysis, we first constructed gene coexpression networks of ConA-induced liver injury using global transcriptome. We identified the brown module with 910 genes and the turquoise module with 2992 genes as the top 2 modules (Figure 3A).

At 3 h after ConA treatment, the brown module showed the highest module significance (MS) value; the turquoise module showed the highest MS value at 24 h after ConA treatment (Figure 3B–3C). Functional enrichment analyses showed that the brown module was significantly enriched in inflammatory response and cytokine-cytokine receptor interaction pathways, whereas, the turquoise module was significantly enriched in the oxidation-reduction process and chemical carcinogenesis pathway (Supplementary Table 2).

We identified the hub genes in the turquoise module using the network feature selection of WGCNA (Figure 3D). The genes in the turquoise module were ranked according to their gene significance (GS) values. Most of the hub genes in the turquoise module overlapped with the top dynamic genes identified by Trendy (Figure 3E). The highest ranked hub gene in the turquoise module was Cluster determinant 63 (*Cd63*, GS = 0.996). *Cd63* is an exosomal marker in the drug-resistant HCC-derived exosomes [16]. *Cd63* and *Cd63*-related genes were enriched in pathways related to chemical carcinogenesis and metabolic pathways (Figure 3F).

### *Cd63* silencing ameliorates ConA-induced hepatic injury in mice

**Figure 4:**
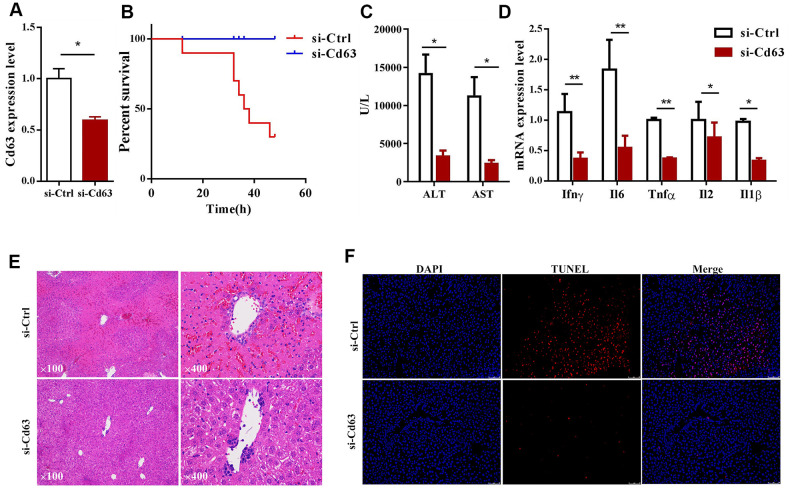
***Cd63* silencing protects against *in vivo* ConA-induced liver injury.** (**A**) qRT-PCR analysis shows *Cd63* mRNA expression in the liver tissues of control (sh-Ctrl) and *Cd63* knockdown (sh-*Cd63*) group mice. (**B**) Survival curves show overall survival rates of sh-Ctrl and sh-*Cd63* group mice after 15mg/Kg ConA treatment. (**C**) Comparison of serum ALT and AST levels in the sh-Ctrl and sh-*Cd63* group mice treated with ConA. (**D**) qRT-PCR analysis shows the relative mRNA levels of pro-inflammatory genes, *IL-1β, IFN-γ, IL-2, IL-6, and TNF-α* in the liver tissues of sh-Ctrl and sh-*Cd63* group mice. All data are shown as means ± SEM (n = 5 per group). * *p* < 0.05; ** *p* < 0.01; *** *p* < 0.001. (**E**) Representative images show H&E stained liver sections of sh-Ctrl and sh-*Cd63* group mice. (**F**) Representative images show TUNEL stained liver sections of sh-Ctrl and sh-*Cd63* mice.

We then investigated the effects of silencing *Cd63* in the ConA-induced hepatitis model mice. The mice injected with lentiviruses carrying *Cd63*-specific shRNAs showed 50% reduction in the *Cd63* mRNA levels in the liver tissues compared to the corresponding controls (Figure 4A). The survival rates of the *Cd63*-silenced ConA-induced mice were significantly higher than the corresponding control group mice (Figure 4B). We also analyzed serum ALT and AST levels in the control and *Cd63*-silenced groups of mice at 8 h after ConA injection. The serum ALT and AST levels, which were estimated at 8 h after ConA injection, were significantly lower in the *Cd63*-silenced group mice compared to the control group mice (Figure 4C).

H&E stained liver sections estimated at 24h showed widespread tissue necrosis in ConA-treated control group mice, but necrosis was significantly reduced in the *Cd63*-silenced group mice treated with ConA (Figure 4E). TUNEL assay results showed that hepatocyte death was significantly reduced in the *Cd63*-silenced group mice treated with ConA compared to the ConA-treated control group mice (Figure 4F). qRT-PCR analysis showed that the mRNA levels of pro-inflammatory mediators such as *IL-1β, IFN-γ, IL-2, IL-6, TNF-α* were significantly lower in the liver tissues from the *Cd63*-silenced group mice compared to the liver tissues from the ConA-treated control group mice (Figure 4D). Overall, our results suggest that *Cd63* deletion significantly reduces liver injury in the ConA-treated mice.

## DISCUSSION

AIH is a complex polygenic disorder that requires development of new effective therapeutic strategies including targeted therapies to reduce morbidity and mortality. Though evidence has showed that several genes including multiple major histocompatibility complex (*MHC*)-related genes, *AIRE* (autoimmunity regulator) and *CYP2D6* (hepatocyte enzyme) are associated with AIH risk [17], but the mechanistic details regarding the development and pathogenesis of AIH are complex and remain to be fully elucidated.

We analyzed dynamic changes in the ConA-induced hepatitis mouse model at individual gene and whole transcriptome levels to identify key genes involved in AIH pathogenesis. We used Trendy software to identify top dynamic genes based on the time-course gene expression data of liver tissue samples from the ConA-hepatitis model mice. We then identified several key gene modules and hub genes using WGCNA. Majority of the hub genes in the turquoise module were the top dynamic genes identified by Trendy. *Cd63* was the top hub gene in the turquoise module. The *in vivo* ConA hepatitis mouse model showed AIH-like pathology. *Cd63* silencing significantly reduced liver pathology and increased survival outcomes in the ConA-treated mice. The role of *Cd63* has not been documented in liver injury. However, several studies show that *Cd63* plays a crucial role in tumor cell plasticity and metastasis [18]. Tissue inhibitor of metalloproteinases-1 (*Timp1*) signaling via *Cd63* activates hepatic stellate cells and creates a favorable environment in the liver for the pancreatic tumor cells [19]. We demonstrate that ConA treatment increases *Timp1* expression at all time points analyzed (Supplementary Figure 2). We also demonstrate that *Cd63* knockdown reduces serum AST and ALT levels as well as pathological lesions in the liver tissues of ConA-treated mice. Previous studies demonstrate that ConA treatment activates immune cells and mediates chronic inflammation resulting in the secretion of several pro-inflammatory mediators that aggravate liver injury [20]. We demonstrate that ablation of *Cd63* significantly reduces the production of pro-inflammatory mediators and necrosis in the liver tissues.

Our study identified several top dynamic genes that might play a significant role in AIH pathology. Future investigations are necessary to unravel the functions of these genes in AIH. For instance, *Saa3* is an inducible form of serum amyloid A (SAA) that is highly expressed in the adipose tissues under acute inflammatory stimuli and obesity, and it promotes monocyte chemotaxis and macrophage accumulation in the adipose tissues [21]. Moreover, *Saa3* is required for normal weight and metabolic functions of the immune system in mice [22]. *Sult2a8* (2810007J24Rik) catalyzes the 7α-hydroxyl sulfation of the bile acids [23], and acts as a novel PPARα-dependent gene [24]. *Slc10a1* (*Ntcp*) functions as a bile acid transporter and prevents bile acid toxicity after partial hepatectomy in mice [25]. Further investigations are necessary to determine if the top dynamic genes are therapeutic targets for AIH.

In summary, our study identifies several candidate genes that are differentially regulated during ConA-mediated hepatitis using Trendy and WGCNA. Furthermore, we demonstrate that ablation of *Cd63* reduces ConA-induced liver pathology and improves survival rates in the ConA hepatitis model mice.

## MATERIALS AND METHODS

### Transcriptome data analysis

The time-course transcriptome profile of the ConA hepatitis murine model was retrieved from the GSE45413 dataset in the GEO database [26]. This dataset was generated with liver tissues collected at 0 h, 3 h, and 24 h after 10mg/kg ConA injection into 8-12 week old C57BL/6 male mice. The microarray data was normalized by selecting probes for genes with a mean expression in the top 75% and removing the lowly-expressing genes. Then, we selected the genes with above average expression variance. We then collapsed different probes that targeted the same gene, resulting in 6936 genes [27]. Finally, we identified differentially expressed genes using the limma R package.

### Identification of top dynamic genes using Trendy

We used the Trendy R package to characterize dynamic gene-specific expression patterns over a time-course during acute liver injury [28]. In brief, Trendy fits a set of segmented regression models with varying numbers of breakpoints for each gene. Each breakpoint represents a dynamic change of gene expression over time. Genes with high R^2^ values were categorized as top dynamic genes. Then, the parameter estimates of the optimal model including the sign and p-value of the slope estimate were used to determine the direction (up, down, or no-change) of the changes in the expression of the top dynamic genes over time.

### Weighted gene co-expression network analysis

The top dynamic genes were evaluated by weighted gene co-expression network analysis (WGCNA) to identify gene modules and hub genes [29]. In brief, the eigengene module was identified based on the weighted average of the gene expression profiles by evaluating the matrix of pair-wise Pearson's correlation coefficients. Then, the gene significance (GS) was computed for each gene within the eigengene module at all time points after ConA injection (3 h and 24 h). The geometric mean was then calculated for the absolute values of all the GS values within each module to determine the module significance (MS) of each module. Modules with higher MS values significantly correlated with the trait (time of liver injury). The network of the module genes was visualized using Cytoscape.

### Functional enrichment analysis

We used the DAVID database (https://david-d.ncifcrf.gov/) [30], and GOplot [31] to determine significant gene ontology (GO) terms and Kyoto Encyclopedia of Gene and Genomes (KEGG) pathways related to the module genes.

### Establishment of *Cd63* knockdown mice

We obtained 8-12 week old C57BL/6 male mice weighing 20-25g from the Shanghai Slac Laboratory Animal Co. Ltd (Shanghai, China). The mice were housed in a specific-pathogen-free facility with a consistent room temperature and humidity. We generated lentiviruses carrying shRNA-*Cd63* (CCAGGTGAAGTCAGAGTTTAA) or control scrambled shRNA (shRNA-Ctrl) vector as previously described [32]. Four weeks before the ConA injection, lentiviruses carrying the sh-*Cd63* or sh-Control (sh-Ctrl) was injected into the tail vein of mice (n=5/group).

### ConA-induced hepatitis model mice

We injected mice with 10 mg/kg ConA (prepared in saline) through the lateral tail vein. For the survival assay, 15 mg/kg ConA was used. At the indicated time points (0 h, 3 h, and 24 h), blood samples were obtained through retro-orbital bleeds and serum samples were prepared and stored at -80° C until measured by automatic biochemical analyzer (Hitachi Auto Analyzer 7170, Japan) for aspartate aminotransferase (AST) and alanine aminotransferase (ALT). Then, the mice were sacrificed and liver samples were harvested and stored for further experiments.

### Quantitative real time PCR

Total RNA was extracted from the murine liver tissues using Trizol (Invitrogen). Equal amounts of RNA were reverse-transcribed into cDNA using the PrimeScript RT reagent kit (Takara Bio). Then, gene expression was analyzed by qPCR with the SYBR Premix Ex Taq kit (Takara Bio) in the ABI 7900 Real-Time PCR System. The expression of various genes relative to *GAPDH* (housekeeping gene) was determined using the 2^-ΔΔCt^ method.

### Histopathology assays

The murine liver tissue samples were fixed in 4% paraformaldehyde for 48 h and then paraffin embedded. Then, 4-5 μm thick paraffin embedded liver sections were cut, deparaffinized with xylene, rehydrated with decreasing concentrations of ethanol, and stained with hematoxylin and eosin (H&E). Cryosections of liver tissues were fixed with 4% paraformaldehyde in PBS for 15 min. Then, relative levels of apoptosis were quantified in all samples using the TUNEL assay with the In Situ Cell Death Detection Kit (Roche) according to the manufacturer's instructions.

### Statistical analysis

The data are expressed as means ± SEM. The statistical differences between samples were compared using 2-tailed Student’s t tests. *p*<0.05 was considered statistically significant.

### Ethical standards

The experiments were carried out according to the protocols approved by the Ethical Committee of the Affiliated Hospital of Hangzhou Normal University (Approval No. 2019(02)-HS-51).

## Supplementary Material

Supplementary Figures

Supplementary Table 1

Supplementary Table 2
