# Supplementary material for: Identification and characterization of dynamically regulated hepatitis-related genes in a concanavalin A-induced liver injury model
Source: Aging (Albany NY). 2020 Nov 18;12(22):23187–99. doi: 10.18632/aging.104089 (PMC7746381; doi:10.18632/aging.104089)
Supplement: Supplementary Figures [file aging-12-104089-s001..pdf]

SUPPLEMENTARY FIGURES

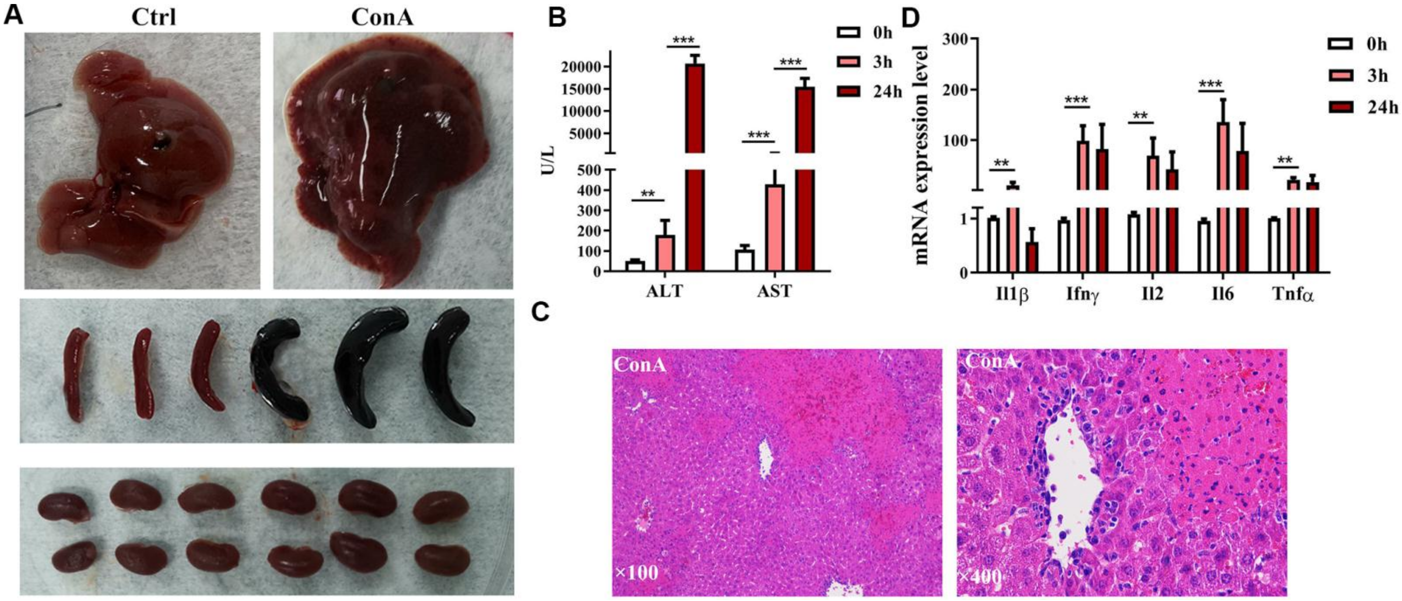

Supplementary Figure 1. Evaluation of liver damage in mice after ConA treatment at 24h. (**A**) Representative images show liver, kidney and spleen of mice injected with 10 mg/Kg ConA or PBS. (**B**) The serum ALT and AST levels in mice treated with 10 mg/Kg ConA or PBS. (**C**) Representative images show H&E stained liver sections in mice treated with10 mg/Kg ConA or PBS. (**D**) qRT-PCR analysis shows relative mRNA levels of pro-inflammatory genes, *IL-1β, IFN-γ, IL-2, IL-6, and TNF-α* in liver tissues of mice treated with10 mg/Kg ConA or PBS. * $p < 0.05$; ** $p < 0.01$; *** $p < 0.001$.

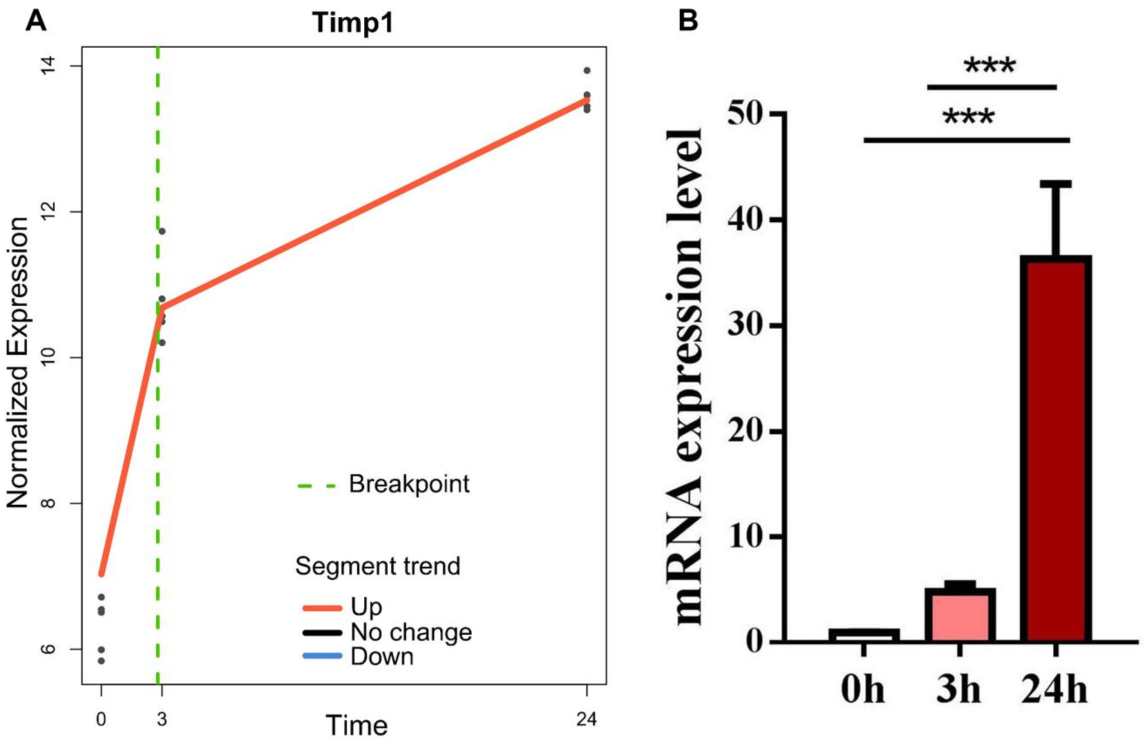

Supplementary Figure 2. *Timp1* expression in mice after ConA treatment. (**A**) Trendy analysis results show the dynamic expression of *Timp1* in ConA-treated mice (3 h and 24 h post-ConA treatment). (**B**) qRT-PCR analysis shows *Timp1* expression in the liver tissues of mice treated with ConA at 0h, 3 h and 24 h post-treatment respectively. *** p <0.001.
